# Pharmacy fall prevention services for the community‐dwelling elderly: Patient engagement and expectations

**DOI:** 10.1111/hsc.13475

**Published:** 2021-06-16

**Authors:** Marle Gemmeke, Ellen S. Koster, Obaid Janatgol, Katja Taxis, Marcel L. Bouvy

**Affiliations:** ^1^ Division of Pharmacoepidemiology and Clinical Pharmacology Utrecht Institute for Pharmaceutical Sciences (UIPS) Faculty of Science Utrecht University Utrecht The Netherlands; ^2^ Department of Pharmacotherapy, Pharmacoepidemiology and Pharmacoeconomics (PTEE) Faculty of Science and Engineering Groningen Research Institute of Pharmacy University of Groningen Groningen The Netherlands

**Keywords:** drug safety, elderly, fall prevention, fall risk‐increasing drugs, patient engagement, perspectives, pharmacy

## Abstract

Medication use is an important risk factor for falls. Community pharmacists should therefore organise fall prevention care; however, little is known about patients' expectations of such services. This qualitative study aims to explore the expectations of community‐dwelling older patients regarding fall prevention services provided by community pharmacies. Telephone intakes, followed by three focus groups, were conducted with 17 patients, who were aged ≥75 years, used at least one fall risk‐increasing drug (FRID) and were registered at a community pharmacy in Amsterdam, the Netherlands. Some time of the focus groups was spent on playing a game involving knowledge questions and activities to stimulate discussion of topics related to falling. Data were collected between January 2020 and April 2020, and all focus groups were audiotaped and transcribed verbatim. The precaution adoption process model (PAPM) was applied during data analysis. Patients who had already experienced a fall more often mentioned that they took precautions to prevent falling. In general, patients were unaware that their medication use could increase their fall risk. Therefore, they did not expect pharmacists to play a role in fall prevention. However, many patients were interested in deprescribing. Patients also wanted to be informed about which medication could increase fall risk. In conclusion, although patients initially did not see a role for pharmacists in fall prevention, their perception changed when they were informed about the potential fall risk‐increasing effects of some medications. Patients expected pharmacists to focus on drug‐related interventions to reduce fall risk, such as deprescribing.

**What is known about this topic?**

- Medication use is an important risk factor for falls.
- Community pharmacists can contribute to fall prevention through the identification and modification of fall risk‐increasing drug (FRID) use in older people.
- Prevention programmes should match with patients' preferences to ensure patient engagement.

**What this paper adds?**

- For older people, the experience of a fall would be the most important reason to engage in pharmacist‐led fall prevention services.
- Most older patients were unaware of the fall risk‐increasing effects of medication. Therefore, they lacked expectations about pharmacy fall prevention services.
- Older patients' interest in pharmacy fall prevention services related primarily to the deprescribing and provision of information about FRIDs.

## 1 INTRODUCTION

One‐third of people aged 65 years and older fall at least once each year (Morrison et al., 2013). Given the potentially serious consequences of falls, including physical injury and increased use of health services, the prevention of falls is of utmost importance (Stel et al., 2004). Furthermore, people who experience a fall incident often develop a fear of falling, which leads to limitations in daily activities with social withdrawal, functional decline and reduced mobility. A fear of falling also increases fall risk (Liu, 2015).

Falling is a multifactorial problem, and medication use is an important, potentially modifiable risk factor (Fonad et al., 2015; Huang et al., 2012). Since one of the core tasks of community pharmacists is to ensure safe medication use and prevent medication‐related problems, they should play a prominent role in reviewing the use of fall risk‐increasing drugs (FRIDs) (Cooper & Burfield, 2009; Walsh et al., 2019). Apart from this, pharmacists can provide information on other modifiable risk factors, such as exercise, diet and a safe home environment.

Prevention programmes should align with patients' preferences to ensure patient engagement. Therefore, the expectations of patients must be taken into account during the development of interventions (Baris & Seren Intepeler, 2019; McMahon et al., 2011). Fall prevention programmes previously failed because of a mismatch between the views of healthcare providers and those of their patients regarding fall risk assessment. Patients did not accept their individual fall risk assessment by nurses (Radecki et al., 2018). Moreover, patients had diverse reasons for not wanting to participate in an exercise‐based fall prevention programme delivered by community care staff (e.g. patients being too busy, already doing exercise, being too old, experiencing a fear of new things or falling and disliking exercise) (Burton et al., 2020). Most importantly, since patients often underestimate their own fall risk, they are not motivated to enrol in fall prevention programmes (Bowling & Ebrahim, 2001; Chen et al., 2016). Furthermore, patients' autonomy must be maintained during such programmes to keep them engaged (McMahon et al., 2011).

Patients' needs and expectations regarding fall prevention programmes delivered by community pharmacies have not been studied before. More knowledge is needed on how patients would like pharmacists to approach them for fall prevention interventions and what the intervention programmes should look like. In this qualitative study, we investigated the engagement of community‐dwelling older people in fall prevention, focusing on fall prevention services conducted by community pharmacies.

## 2 METHODS

### 2.1 Study setting and population

A qualitative study was conducted consisting of short individual telephone intakes followed by focus group discussions. One researcher (MG) selected patients from the pharmacy information system of a community pharmacy in Amsterdam, and another researcher (OJ) invited them to participate in the focus groups.

The following inclusion criteria were used for selection of patients:

- Age ≥75 years;
- Simultaneous use of at least five drugs, with at least one being a FRID (either cardiovascular or psychotropic) (Seppala, van de Glind, et al., 2018; Seppala, Wermelink, et al., 2018; de Vries et al., 2018);
- Community‐dwelling;
- Physically and mentally able to attend the focus group in the community health centre;
- Proficient in Dutch.

Patients were invited by telephone, and after verbal consent, a telephone intake followed. They were briefly asked about their fall experiences and interest in fall prevention (see below). Thereafter, an information letter and consent form were sent by postal mail to their addresses. All participants provided written informed consent before the start of the focus group discussions. All data were collected between January 2020 and April 2020.

The study was approved by the institutional review board of the Division of Pharmacoepidemiology and Clinical Pharmacology, the Department of Pharmaceutical Sciences, Utrecht University. Results were reported according to the consolidated criteria for reporting qualitative research (COREQ) guidelines (Supporting Information, Appendix S1) (Tong et al., 2007).

### 2.2 Telephone intakes

Semi‐structured telephone intakes of approximately 30 min were performed with participants prior to conducting the focus groups. These intakes aimed to obtain individual fall‐related background information, such as previous fall experiences, applied precautions to reduce fall risk and interest in pharmacy fall prevention services. The researcher (OJ) used a topic list (Table 1) for the telephone intakes and completed a structured form immediately after each intake.

**TABLE 1:** The topic list used in the telephone intakes and the topics and statements addressed during the focus groups

	Telephone intakes
Topic	Examples of questions
Fall experiences	*Did you fall in the past?*
	*Are you afraid of falling?*
Precautions	*What are your solutions to reduce fall risk?*
Interest in fall prevention service	*Are you interested in a fall prevention programme from pharmacists?*
Focus groups	
Topic	Examples of questions
Fall experiences	*Did you fall in the past, and are you afraid of falling?*
Precautions	*What are your solutions to reduce fall risk?*
Needs and wants	*What are your needs for fall prevention services in general?*
	*What are your experiences with fall prevention services from other healthcare providers?*
Expectations from pharmacists	*How could pharmacists contribute to fall prevention in your opinion?*
	*What do you expect from pharmacists in fall prevention?*
Topic	Statements
Precautions	*I make sure there are no objects on the floor to prevent them from stumbling over them*.
Interest in fall prevention service	*I am interested in fall prevention services by pharmacists*.
Expectations from pharmacists	*My pharmacist should inform me, when I start using a new drug, about potential fall risk‐increasing adverse effects*.
	*My pharmacist should ask me regularly, preferably every 3 months, about my recent fall history*.
	*My pharmacist should help me with finding solutions I can do myself to reduce my fall risk, including environmental adjustments (e.g. removing carpets, sufficient lighting)*.
	*My pharmacist should inform me about calcium and vitamin D intake to strengthen my bones*.
	*My pharmacists should inform me about mobility and balance exercises to stay fit and vital*.
Deprescribing	*I think one or more of the drugs I use can be discontinued because I am using them daily for long time now*.
	*I wish my pharmacist checks, in agreement with me, which of my drugs increase fall risk and whether I still need them*.
Information about fall prevention/drugs	*Statement 1: I search for information on the internet about solutions to reduce my fall risk*. *or* *Statement 2: I ask my healthcare provider for tips and recommendations to reduce my fall risk*.
	*When I am dizzy and I think my medication caused this, I prefer reading patient information leaflet to consulting my pharmacist*.

Abbreviation

PAM, precaution adoption process model.

### 2.3 Focus groups

Participants were divided into three focus groups, resulting in five to seven participants per session. The duration of each session was 1.5–2 hr. The first focus group was chaired by an experienced pharmacy practice researcher (EK), while two another researchers (MG and OJ) were second listeners, who occasionally stimulated group discussion and took field notes. The second and third focus groups were chaired by OJ, while MG was the second listener during these focus groups and EK took field notes during the second focus group. All focus groups were audiotaped and transcribed verbatim afterwards, and all patients received a short report with the main findings of the focus groups. Data saturation was discussed after the third focus group.

A topic list was made to guide the focus groups (Table 1). First, the findings from the telephone intakes were briefly discussed in the focus groups. Thereafter, additional topics derived from findings of the intakes, the first focus group session and the literature (Table 2), were addressed in those groups.

**TABLE 2:** Scientific foundation of topics addressed during interviews and focus groups

Topic	Scientific foundation
Fall experiences	Acceptance of fall risk impairs the personal identities of older patients (Gardiner et al., 2017). However, by experiencing a fall, personal fall risk may be acknowledged (McInnes et al., 2011). Therefore, previous fall experiences trigger behavioural changes and engage patients in fall prevention activities (Robson et al., 2018)
Precautions	The importance of being careful is often recognised by older people. They avoid certain activities, and precautions are taken, even by patients who deny experiencing a fear of falling (Gardiner et al., 2017). Exploration of the precautions taken provides information about the established engagement in fall prevention
Interest in fall prevention service	Patients have reported that the necessity of fall prevention activities is associated with ageing. It may be disturbing for older patients to belong to the group who is in need of these activities (McInnes et al., 2011). Their interest in a fall prevention service indicates whether they are already engaged
Needs and wants regarding fall prevention service	Older people may experience asking for help in fall prevention as a loss of their independence. However, a fall can seriously impair their independence (Gardiner et al., 2017). When patients recognise that prevention services could also protect their independence, this could enhance their engagement
Expectations from pharmacists	Patients often do not know who should be approached for support in fall prevention (Robson et al., 2018). When they are unaware that their pharmacist could be consulted, it is unlikely that they will ask for the pharmacist's assistance. Therefore, higher established expectations from pharmacists could be related to enhanced patient engagement
Deprescribing	Deprescribing aids in the prevention of adverse drug reactions, including increased fall risk. It has been reported that patients sometimes think their medication might no longer be necessary for the treatment of their disease(s) (Reeve et al., 2013b; Reeve, Wiese, Hendrix, et al., 2013). Therefore, many may be interested in deprescribing and would like to know more about its advantages and disadvantages. Pharmacists can facilitate the deprescribing process, for example, by conducting medication reviews
Information about fall prevention/drugs	For behavioural changes, the understanding of fall risk is essential. Patients are often unaware of potentially modifiable risk factors (Robson et al., 2018). Enhanced patients' knowledge contributes to patient engagement in fall prevention

The group discussion was followed by a game of DobbelFit (‘DobbelFit | Valpreventie | VeiligheidNL’, n.d.). The DobbelFit game—created by VeiligheidNL, a Dutch organisation that aims to prevent accidents and improve safety nationwide—has been developed for healthcare professionals to play together with patients. During the game, patients are challenged to perform simple exercises to improve their balance. Furthermore, the game contains a quiz element with questions on issues such as potential fall risk factors, the benefits of calcium and vitamin D supplementation and medication‐related fall risk. The game was adapted for the focus groups by removing non‐pharmacy‐related questions and by reducing the number of exercise challenges. In the second and third focus groups, the number of knowledge questions was also reduced and replaced by statements about fall prevention. These statements (Table 1) were included to enhance data collection.

### 2.4 Data analysis

All audio recordings of the focus groups were transcribed verbatim. The intake forms and focus group transcripts were imported into NVivo Version 12 software, and participants' names were replaced by a study code to ensure their anonymity. The transcripts were coded independently by two researchers (OJ and MG), and discrepancies in coding were discussed with EK until consensus was reached. Deductive coding was used—the codes were based on the topic list. A number of additional codes were identified during transcription (inductive coding).

### 2.5 Interpretation of the data

The precaution adoption process model (PAPM) was used in the data analysis (Weinstein & Sandman, 1992). This model has often been used to describe patients' decision‐making processes in a wide range of situations, including HPV vaccination (Barnard et al., 2017; Shapiro et al., 2018), treatment for osteoporosis (Adami et al., 2020) and the screening of diverse cancers (Ferrer et al., 2011; Marlow et al., 2017). The PAPM consists of seven stages, representing all stages of taking precautions to reduce risk, and it was considered as the most appropriate model to assess fall preventive health behaviour. In contrast to other health behaviour theories and models, the PAPM includes the stage at which patients are not yet aware of a threat or a risk. In the case of fall prevention, this applies to patients who are not afraid of falling and therefore have not (yet) taken precautions. The PAPM also investigates behavioural changes and patients' reasons for engaging.

## 3 RESULTS

### 3.1 Background characteristics

**FIGURE 1:**
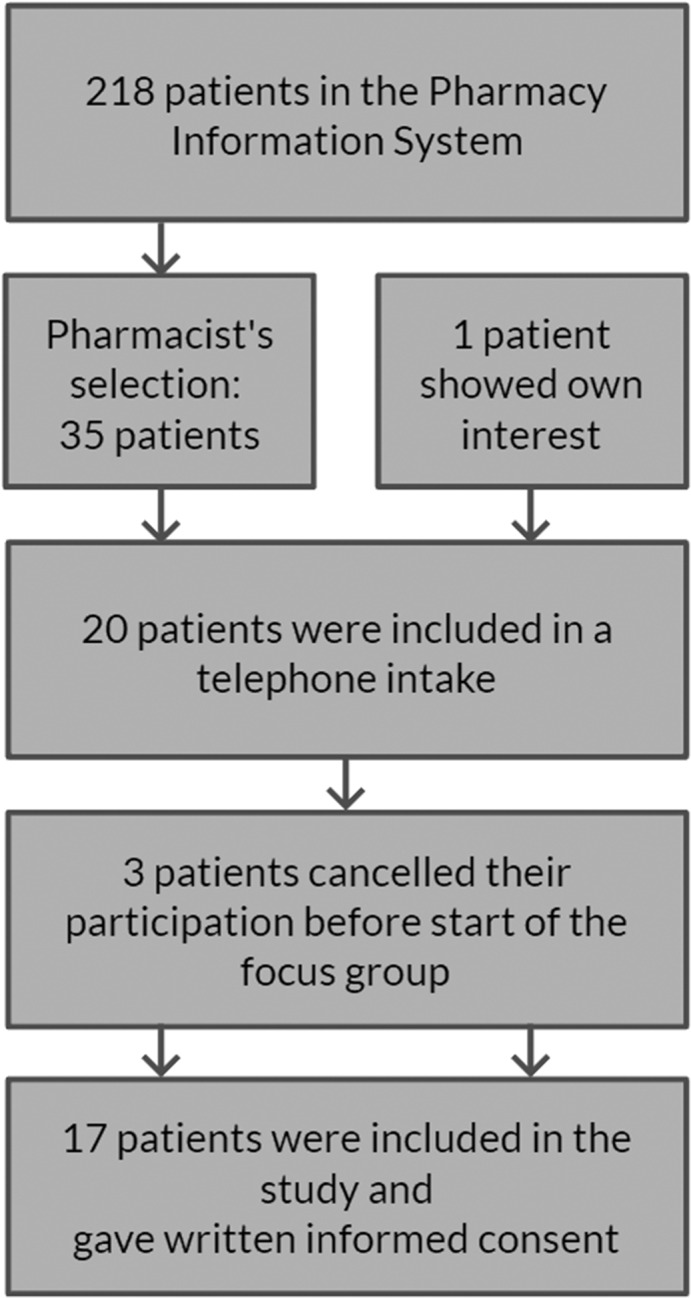
Flowchart of the inclusion of patients in the study

In total, 218 patients aged 75 years or older using five chronic medications were identified from the pharmacy information system. Of these, 35 patients were purposely selected by the researcher/pharmacist (MG) and invited to participate. The reason for this selection was that they were known to visit/contact the pharmacy regularly and were thus able to independently attend the focus group session in the community health centre. Twenty participants agreed to participate, but just before start of the focus groups, three of them cancelled. Therefore, 17 participants attended the focus groups (Figure 1). The reasons for cancellation were having other appointments and not feeling well enough. All participants met the inclusion criteria, except for one woman of 69 years. Her husband, who met the inclusion criteria, was originally invited, but she participated instead of him. This woman's views were comparable with the overall findings, and she had experienced multiple falls.

Slightly more women (52.9%) than men participated, and the mean age of the participants was 82.1 years (standard deviation [sd] = 4.9 years). Most participants (58.8%) reported at least one fall incident (Table 3). During the third focus group, no new topics were addressed, and the research team concluded that data saturation was achieved.

**TABLE 3:** Background characteristics of the patients in the focus groups and telephone intakes

	Patients *N* = 17
Female gender (*N*, %)	9 (52.9%)
Age in years (mean [sd])	82.1 [4.9]
Multidose drug dispensing system (*N*, %)	4 (23.5%)
≥ 1 fall experience(s)^a^ (*N*, %)	10 (58.8%)
Number of dispensed medications (median [Q1–Q3])	8 [6–9]
Number of dispensed FRIDs (median [Q1–Q3])	3 [2–5]

Abbreviation

FRID, fall risk‐increasing drug; *N*, number; Q1, first quantile; Q3, third quantile; sd, standard deviation.

^a^ An estimation of the past 10 years on the basis of what patients said during the intakes and focus groups.

### 3.2 The PAPM

The PAPM consists of seven stages of patients' decision‐making to act on fall prevention. Stage 1 (unawareness) and Stage 2 (non‐engagement) of the model were combined in the analyses, as both describe stages in which patients are not taking precautions to prevent a fall. Stage 3 (undecided about acting) refers to the decision‐making between acting and non‐acting on fall prevention, and Stage 4 (decided not to act) represents non‐acting behaviour. Stage 5 (decided to act), Stage 6 (acting) and Stage 7 (maintenance) describe acting behaviour and were also combined during analyses. Furthermore, the PAPM stage transitions were identified and analysed.

**FIGURE 2:**
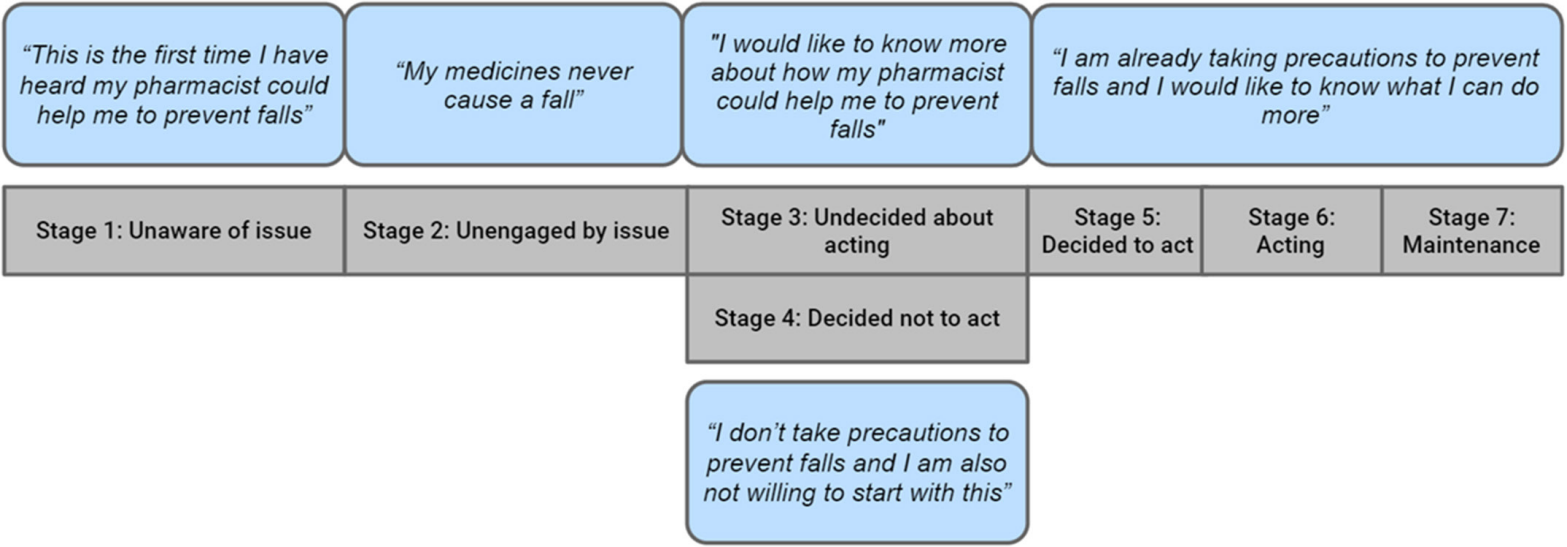
The application of the precaution adoption process (PAPM) model on possible thoughts or beliefs of patients during their decision‐making in fall prevention

Participants were in different stages of the PAPM (Figure 2). Furthermore, they were sometimes found in one PAPM stage for certain behaviours, but in different stages for other behaviours. Table 4 summarises participants' views on the main codes and the related PAPM stages from the focus groups and intakes.

**TABLE 4:** Participants' views on topics

PAPM stage	Codes	Responses *N* = 17
Unaware	No interest in service	Follows directions of drug use carefully without problems (*N* = 1)
	Medication and fall risk	Indicates that medication use did not cause the fall(s) (*N* = 7)
	Deprescribing wants	Believes withdrawal is unnecessary in cases without complaints (*N* = 1)
Unengaged	No interest in service	Perceives no fall risk and is therefore not interested (*N* = 4)
Undecided about acting	Interest in service	Shows interest and wants to know more (*N* = 7)
	Medication and fall risk	Has doubts about how a pharmacist could help (*N* = 4)
	Information search	Looks for information on the World Wide Web, and in magazines, or consults friends/family (*N* = 9)
		Reads patient information leaflet (*N* = 7)
		Consults general practitioner or pharmacist (*N* = 8)
Decided not to act	No interest in service	Believes pharmacy employees are not capable enough (*N* = 1)
	Deprescribing wants	Believes his/her medication is necessary and cannot be withdrawn (*N* = 3)
Decided to act	Interest in service	Clearly displays interest in service (*N* = 4)
	Deprescribing wants	Hopes/wants medication to be withdrawn (*N* = 10)
Acting	Precautions	Is already taking precautions (home safety, walking aid, avoidance of certain activities) (*N* = 14)
Stage transitions	Fall anxiety	Reports fall anxiety (*N* = 5)
		Not afraid, but careful (*N* = 6)

Note

PAPM, precaution adoption process model.

### 3.3 Unawareness and non‐engagement (PAPM Stages 1 and 2).

Patients' perceived fall risk seemed to influence their engagement in fall prevention activities; specifically, a low perceived fall risk was often co‐reported with a low interest in fall prevention. Four patients perceived no risk of falling and were consequently not interested in participating in fall prevention programmes. Those who were not interested in fall prevention services also indicated that they were not taking precautions to reduce fall risk. They stated that they were healthy, exercised and/or walked a lot. Although exercising could be seen as a precaution to prevent falls, these patients explicitly mentioned that they were not taking precautions to prevent falls. One patient who perceived no risk even expected that healthcare providers would agree that he was not at risk:

I don't think pharmacist employees feel the need to ask me about these things [recent fall incidents].

Man, 84 years (Patient 4)

Cognitive pharmaceutical services (CPS; Strand et al., (2012)) are pharmaceutical services that offer provision of information and counselling to enable patients to take responsibility for their own care and correct medication use. Although many patients were positive about such CPS for older people, some patients had doubts about how pharmacists could contribute to fall prevention. They were also surprised that they were approached by the pharmacy to participate in this research:

You are the first pharmacy employee who is asking me about this. But I'm interested in all kinds of advice. However, I don't have any fall experiences.

Man, 84 years (Patient 4)

Many patients were unaware of the fact that medication use could increase fall risk. This was also seen during the DobbelFit game. Participants' understanding of fall‐related drug side effects varied: Some patients had little understanding, while others were able to relate side effects to fall risk. This was reflected in patients' answers to the focus group moderator's question regarding whether diuretics and hypnotics could increase fall risk:

I use diuretics, and because of that, I have to pee five times in a night. But I don't think this increases risk of falling.

Woman, 83 years (Patient 7)

When the blood pressure decreases, this is possible. That's my gut feeling; I am not an expert.

Man, 76 years (Patient 10)

Yes, when blood pressure decreases, you can become dizzy. But I don't fall because of that.

Woman, 83 years (Patient 7)

### 3.4 Undecided about acting (PAPM Stage 3)

Patients in this stage were undecided about acting on fall prevention. Informing them about fall risks seemed to aid in the decision‐making process. Patients would like to receive more attention and appreciated receiving information from pharmacists about the potential fall risk‐increasing effects of drugs:

Yes, [informing about fall risk‐increasing drug effects] is definitely a good thing. It is part of prevention, and therefore, it is good. Yet, I don't know what I will do with the information.

Man, 84 years (Patient 17)

Most patients stated that they primarily tried to solve health‐related problems by themselves. They would search the internet for information about fall prevention or drugs. Articles in popular press were valued as well. They would subsequently consult relatives, neighbours or friends. Only when patients could not solve healthcare problems on their own they would consult a healthcare provider:

First, I would try to investigate the problem on my own. When this does not work, I ask someone who is having the same problem as me, and I ask how he is experiencing it. […] When I cannot solve it myself, then I approach a healthcare provider.

Man, 86 years (Patient 11)

Some patients said that they read patient information leaflets when they received the initial dispensing of a new drug. They expected that patient information leaflets contained relevant information about the fall risk‐increasing side effects of drugs:

When I experience side effects such as dizziness, I would read the patient information leaflet instead of consulting the pharmacy. For example, it is 10 PM and I feel dizzy due to medication, then I read the patient information leaflet. […] It is written by an expert.

Man, 82 years (Patient 16)

However, patient information leaflets were not appreciated by all participants. The abundant description of side effects and the small font size caused some patients to immediately throw those leaflets into the bin. They had a preference for leaflets with a larger font size and more succinct information.

Furthermore, patients were undecided or doubtful about pharmacy fall prevention services. Many patients emphasised the role of the general practitioner (GP) in keeping them well informed. They often preferred to consult their GP first about fall prevention as well as about drug information:

When I feel dizzy, I won't approach the pharmacy, but the general practitioner. […] Even when my drugs cause my dizziness…

Man, 83 years (Patient 8)

### 3.5 Decided not to act (PAPM Stage 4)

Although many patients considered that part of their medication was superfluous, not all patients were interested in deprescribing. They either believed that in the absences of drug complaints, withdrawal efforts were unnecessary or believed their medications were essential to treat their disease(s):

I have never been recommended this [deprescribing medication], since I cannot miss anything. I have a stent in my heart. I have thyroid problems. I need to use antihypertensive drugs.

Woman, 83 years (Patient 7)

Although patients were positive regarding pharmacists regularly asking about recent fall incidents, they did not expect or want to receive lifestyle recommendations from pharmacists. Furthermore, patients mentioned receiving limited attention from pharmacists and hence thought that pharmacists would not have enough time to organise fall prevention care:

I think it would be positive [pharmacists making recommendations about home safety], but every day hundreds of patients are entering the pharmacy. Will they be able to ask about it every time? I can't picture that.

Man, 82 years (Patient 16)

Apart from pharmacists, patients also experienced receiving limited attention from doctors, including GPs. A few patients thought there might even be a relationship between age and the efforts of healthcare providers. When patients experience limited attention, it may hold them to continue consulting their healthcare providers about fall prevention:

I have this feeling that there is not a lot of interest. When I enter the GP practice, I see her looking at the clock. And this is in particular the case with elderly.

Man 84 years (Patient 17).

### 3.6 Acting (PAPM Stages 5, 6, and 7)

Engagement with fall prevention was particularly evident in patients who were already taking precautions. For patients who had experienced a fall, precautions were related to the cause of the fall (e.g. careful on stairs when having fallen from stairs). Precautions most often focused on improving home safety and included the following: removing obstacles from the floor to keep the house neat, covering sharp edges with softer material and avoiding walking in socks or slippers. Other precautions were also mentioned, such as avoiding certain activities, use of a walking aid and participating in a community centre fall prevention programme:

I participated in a fall prevention programme of the community centre. I learned not to walk with hands in pockets on the street, so you can always catch yourself when you fall. It was very good and interesting.

Woman, 81 years (Patient 2)

I don't cycle anymore because of that problem. I would not like to get hospitalized again.

Woman, 79 years (Patient 9)

On the other hand, several patients perceived being at low risk of falling because of their daily exercises. All patients emphasised that daily exercises were important for their overall health status and for maintaining their fitness. Therefore, daily exercise alone could also be seen as some form of engagement with fall prevention:

[…] I landed like a frog on the floor on my both feet and hands. I did not break anything. I was only a little hurt. That was because I exercise. When you are stiff you are more likely to break something.

Woman, 81 years (Patient 2)

Apart from the precautions, most patients also said that they would like their medication to be reviewed. Some patients already even hoped that some medication could be withdrawn. In their opinion, the pharmacist could play an important role here:

I'm using the same medicines for over 25 years now and I think half can be withdrawn… […] The pharmacist and cardiologist should collaborate and think of a sort of drug tapering system for me.

Man, 85 years (Patient 6)

### 3.7 PAPM stage transitions

PAPM stage transitions were often triggered by the experience of a fall. Patients who had frequently fallen had developed fall anxiety or were more careful. A woman started taking precautions (e.g. using a walking cane, going out for a walk less) after she had experienced a fall:

I am very busy, and I am member of many committees. […] Since my pelvic fracture, I am afraid to fall again. I used to walk to the square back and forth, but I don't do that anymore.

Woman, 88 years (Patient 3)

At that time, she was possibly unaware that her decision to avoid activities for fear of falling may lead to functional decline and subsequently increased fall risk. In the telephone intake, this woman was highly engaged; she mentioned being interested in all forms of help to prevent falls because she did not want to fall again. Furthermore, another patient experienced fall anxiety after a fall and consequently adapted his home environment:

I am a little afraid of falling after I fell. I removed the carpets straight away. […] I have laminate flooring now.

Man, 85 years (Patient 6)

As noted, patients were often unaware about the fall‐related side effects of medication. Hence, with regard to this topic, they were found in PAPM Stage 1. However, some indicated that informing them about these effects would trigger them to engage in deprescribing, corresponding to PAPM Stage 5:

When the pharmacy tells me I lose balance due to medication, then I would ask for an alternative.

Man, 86 years (Patient 11)

## 4 DISCUSSION

Patients are at different stages of engagement in in fall prevention activities, ranging from being unaware of fall risks to being highly active in the prevention of falls. Therefore, they have different needs and expectations. In particular, patients who had previously experienced a fall were more inclined to prevent future falls and displayed interest in pharmacy fall prevention services.

Our findings confirm previous results demonstrating that older patients often underestimate their fall risk and are therefore not engaged in fall prevention activities (Bowling & Ebrahim, 2001; Chen et al., 2016; Yardley et al., 2006). Furthermore, it has been reported that patients who have experienced a previous fall are more inclined to acknowledge their fall risk (McMahon et al., 2011).

Regardless of the stage of engagement, patients were unaware of the existence of FRIDs. Fall risk as an adverse effect of medication was often not acknowledged by patients, and it seemed to impact the level of engagement in a pharmacy fall prevention service. In the literature, patients' belief that their medication is necessary and beneficial is an important barrier for deprescribing (Reeve et al., ,^2013^, 2013a). In our study, a few patients also mentioned the necessity of medication, and this was served as an argument to not be engaged in a medication review focused on reducing fall risk.

Patients wished to be informed by the pharmacist about how their medication use may increase their fall risk (e.g. at the first dispensing of a new drug). They also expected patient information leaflets to contain this information. Our findings correspond with earlier findings that patients are positive about being educated about their safety. Despite this, informing patients might not always be sufficient for actual behavioural changes (Schwappach, 2010).

From the patient perspective, pharmacists' fall prevention interventions should focus on deprescribing and providing information about how medication may enhance fall risk. Informing patients could facilitate engagement when they are in PAPM Stage 1 or 2 (unawareness/non‐engagement) and support their decision‐making when they are in PAPM Stage 3. Many patients in our study were also interested in targeted interventions, which suggests that these patients were already in PAPM Stage 5 (decided to act). Specifically, these patients indicated being interested in deprescribing. They may be concerned about the high number of drugs, wondering whether all drugs were still necessary. Additionally, it has been shown that patients' drug knowledge is often poor, but crucial for involvement in decision‐making (Modig et al., 2012). Deprescribing interventions presumably will be more successful when patients have increased drug risk awareness. Earlier findings suggest that when patients are not experiencing side effects and are not concerned about future harm, they may not see the benefit of drug withdrawal (Reeve et al., 2016). However, a previous study also found that over 90% of older patients would like to try medication withdrawal, as long as the prescriber agrees (Reeve, Wiese, Hendrix, et al., 2013). This corresponds to our findings: Although not all patients were engaged in fall prevention in general, many still showed interest in deprescribing.

Patients who had experienced a fall tended to acknowledge their fall risk more often and were consequently more frequently found in PAPM Stage 5, 6 or 7 than the others. As a side note, PAPM stages were not consistent for all aspects of fall prevention activities, as individual patients were sometimes found in different PAPM stages for different fall prevention activities. Overall, these patients were consciously adapting precautions, including reducing home environmental hazards, avoiding outdoor activities (walking, cycling) and using a walking aid (e.g. walking stick or walker). Although most of these precautions were helpful in preventing falls, avoidance of activities can have adverse effects. A strong fear of falling has been associated with functional decline, social withdrawal, decreased quality of life, increased risk of falling and institutionalisation (Liu, 2015). Thus, the adapted precautions because of fall anxiety may not always be beneficial for fall prevention. On the plus side, a fear of falling indicates patients are more or less engaged and hence should at least be found in PAPM Stage 3.

Patients were sceptical about whether pharmacists could organise fall prevention, mentioning that pharmacists and other healthcare providers do not have enough time to do so. Furthermore, because of limited time, they expected pharmacists to focus primarily on medication safety. Despite this, patients reported that they would like to receive more attention from their health care providers.

### 4.1 Strengths and limitations

An important strength of this study was the combination of the telephone intakes and focus group which provided comprehensive data. The telephone intakes ensured that the perspectives of all patients, particularly those who were more reluctant to speaking in groups, were investigated. Data from the intakes were used as input for the set‐up of the focus groups. In these groups, patients were encouraged to respond to discussions or complement one another's opinions. In particular, the use of the DobbelFit game during the focus groups was innovative, contributed to a relaxed atmosphere and was appreciated by the participants. The PAPM supported the data analyses, as it helped to identify the stages and engagement triggers of patients. Despite the PAPM being applied retrospectively, during data analysis, the model fitted the data well and enhanced interpretability.

The major limitation of this study was the generalisability of findings. First, all participants were from one single pharmacy in the suburb area of Amsterdam. However, the organisation of healthcare may differ in a strongly urbanised environment compared with small villages. It is challenging for healthcare providers to establish strong relationships with patients in the larger healthcare centres of cities. Therefore, satisfaction about healthcare is generally higher in rural populations (Batbaatar et al., 2017). Since patients' ideas about strong relationships with healthcare providers might differ in a village, their needs and expectations about health services, including fall prevention, might also differ. Second, participants needed to be able to visit the pharmacy. Therefore, the frailest patients with physical disabilities were not included in our study. Third, only polypharmacy patients were included. However, deprescribing may also be relevant for patients who are using FRIDs but do not fall into the polypharmacy category. Fourth, because participants needed to be able to communicate in Dutch, all participants were native Dutch speakers. However, differences could be expected among patients from ethnic minorities. Since their primary healthcare use and health literacy may differ, they would possibly engage less with pharmacists and may have an impaired ability to find and understand fall prevention information (van der Gaag et al., 2017). Fifth, the focus group design might have led to an over‐representation of the views of more dominant participants. For this reason, the focus group moderators attempted to allow all participants to raise their voices. Lastly, our study has not repeated some subgroup viewpoints demonstrated in previous studies. For example, previous studies found that a fear of falling, and subsequent engagement in fall prevention, was also found in patients without fall experiences (Scheffer et al., 2008). Furthermore, another subgroup has also been identified in studies, but not in our work. This group covers patients with many fall experiences but who consider themselves to be ‘non‐fallers’ and who neither experience fall anxiety nor are engaged in fall prevention (Gardiner et al., 2017). With the exception of those viewpoints, our findings correspond to earlier findings from other studies, which strengthens the idea the perspectives are applicable to most patients.

### 4.2 Implications

Pharmacists should spend more time on fall prevention (e.g. screening for patients at risk and informing them about fall prevention). For example, it could be part of medication reviews, and pharmacists should inform patients about the risk of using a FRID at first dispensing. Patients could then engage in fall prevention, and their awareness about fall‐related drug risks would increase. Pharmacists should focus particularly on deprescribing interventions to reduce fall risk in older patients. For risk factors other than medication use, pharmacists could inform and refer patients to other healthcare providers; they should hence collaborate with GPs and other healthcare providers, which is a recommended approach for successful fall prevention (Kobayashi et al., 2017).

Pharmacy fall prevention care should specifically be provided to patients using FRIDs and those who have reduced mobility (e.g. patients who are using a walking aid or standardly request their medication to be home‐delivered). Pharmacists could consider organising educational group sessions about fall prevention for these patients. In these sessions, evidence‐based effective interventions should be addressed, including the deprescribing of FRIDs (Blalock et al., 2010; van der Velde et al., 2007), the relevance of exercising and home environmental recommendations (Gillespie et al., 2012).

In addition to informing patients orally or in group sessions, providing written information should be adequate as well. Patients most often preferred to read or search for information about falls and drugs themselves rather than consulting their healthcare provider. A previous study revealed that patients were passive in consulting their caregiver, because they thought their health professionals would inform them if there was a problem. In contrast, caregivers often mentioned being reactive in providing information (Lee et al., 2013). Encouragement from health practitioners is important for patients to participate in fall prevention activities (Yardley et al., 2006). Therefore, the information provided in patient information leaflets should be complete, with a section on fall‐related side effects. Future research should investigate whether educating patients on the relationship between medication and fall risk increases their engagement in fall prevention services offered by pharmacists.

## 5 CONCLUSION

Although patients were initially doubtful about the role of pharmacists in fall prevention, this changed when they were informed about the potential fall risk‐increasing effects of some medications. Interest came mainly from patients who had experienced a fall. Furthermore, patients expected pharmacists to focus on drug‐related interventions to reduce fall risk, such as deprescribing. Finally, patients wanted to be well informed, both orally and in writing, about FRID effects.

## CONFLICTS OF INTEREST

There were no conflicts of interest.

## AUTHOR CONTRIBUTIONS

*Marle Gemmeke:* Conceptualisation, Investigation, Data Curation, Writing – Original Draft. *Ellen Koster:* Conceptualisation, Investigation, Supervision, Writing – Review & Editing. *Obaid Janatgol:* Conceptualisation, Investigation. *Katja Taxis:* Supervision, Writing – Review & Editing. *Marcel Bouvy:* Supervision, Writing & Editing.

## Supporting information

Appendix S1

## Data Availability

Author elects to not share data due to privacy/ethical restrictions.
