# Supplementary material for: Pharmacy fall prevention services for the community‐dwelling elderly: Patient engagement and expectations
Source: Health Soc Care Community. 2021 Jun 16;30(4):1450–61. doi: 10.1111/hsc.13475 (PMC9290894; doi:10.1111/hsc.13475)
Supplement: Supplementary file 1 — Appendix S1 [file HSC-30-1450-s001.docx]

## Appendix S1: Consolidated criteria for reporting qualitative studies (COREQ): 32-item checklist

| No | Item | Guide questions/description | Check? |
| --- | --- | --- | --- |
| **Domain 1: Research team and reflexivity** | | | |
| Personal Characteristics | | | |
| 1. | Interviewer/  facilitator | Which author/s conducted the interview or focus group? | The telephone intakes were conducted by OJ and focus groups were conducted by OJ, MG and EK. |
| 2. | Credentials | What were the researcher's credentials? E.g. PhD, MD | The credentials can be found in the authors’ list. |
| 3. | Occupation | What was their occupation at the time of the study? | OJ was a master student pharmacy, MG was a specialist in community pharmacy and PhD student, EK was an experienced researcher. |
| 4. | Gender | Was the researcher male or female? | OJ was male, MG and EK were female. |
| 5. | Experience and training | What experience or training did the researcher have? | EK was experienced with interviews and guiding focus groups, while OJ and MG were not experienced with focus groups. |
| Relationship with participants | | | |
| 6. | Relationship established | Was a relationship established prior to study commencement? | MG and OJ were both employed in the pharmacy. Therefore, with some patients a relationship was established prior to the study. Furthermore, OJ personally invited patients by telephone and had built a relationship during this conversation of approximately 30 minutes before the patients attended the focus groups. |
| 7. | Participant knowledge of the interviewer | What did the participants know about the researcher? e.g. personal goals, reasons for doing the research | OJ introduced himself at telephone invitation and informed the participants about the research goal. Before start of the focus groups the research team introduced themselves and informed the patients again. The patients were also informed by a patient information letter enclosed to the informed consent form. |
| 8. | Interviewer characteristics | What characteristics were reported about the interviewer/facilitator? e.g. Bias, assumptions, reasons and interests in the research topic | MG was a pharmacist and OJ was a master pharmacy student. Their interest was how a future fall prevention service by pharmacists could be implemented, which corresponds to the wishes of the(ir) patients. Also, they were interested whether the DobbelFit could be recommended to pharmacists to play with their patients to enhance engagement for fall prevention. |
| **Domain 2: study design** | | | |
| Theoretical framework | | | |
| 9. | Methodological orientation and Theory | What methodological orientation was stated to underpin the study? e.g. grounded theory, discourse analysis, ethnography, phenomenology, content analysis | The Precaution Adoption Process Model was used to analyse the data and underpin the study. |
| Participant selection | | | |
| 10. | Sampling | How were participants selected? e.g. purposive, convenience, consecutive, snowball | Participants were selected on basis of the Pharmacy Information System (PIS). Pharmacist (MG) made a pre-selection of patients. This pre-selection was mainly based on patients of who was known they were able to visit the pharmacy and who were able to communicate in Dutch. |
| 11. | Method of approach | How were participants approached? e.g. face-to-face, telephone, mail, email | The participants were approached by telephone. |
| 12. | Sample size | How many participants were in the study? | 17 participants were included in the entire study. |
| 13. | Non-participation | How many people refused to participate or dropped out? Reasons? | 3 participants cancelled their participation: 2 participants planned another activity at the same time and 1 participant was not feeling well. |
| Setting | | | |
| 14. | Setting of data collection | Where was the data collected? *e*.g. home, clinic, workplace | The data were collected in a room of the community health centre at the location of the pharmacy. |
| 15. | Presence of non-participants | Was anyone else present besides the participants and researchers? | There were no-other attendees besides the participants and researchers. |
| 16. | Description of sample | What are the important characteristics of the sample? e.g. demographic data, date | The background characteristics of the participants are reported in Table 1. All participants were residents of Amsterdam. |
| Data collection | | | |
| 17. | Interview guide | Were questions, prompts, guides provided by the authors? Was it pilot tested? | The questions, prompts and guides were provided by the authors, but not pilot tested. |
| 18. | Repeat interviews | Were repeat interviews carried out? If yes, how many? | There were no repeat interviews. |
| 19. | Audio/visual recording | Did the research use audio or visual recording to collect the data? | The research team used audio-recording during the focus groups. |
| 20. | Field notes | Were field notes made during and/or after the interview or focus group? | Field notes were made during the intakes and focus groups. |
| 21. | Duration | What was the duration of the interviews or focus group? | The durations of the intakes and focus groups were respectively approximately 30 minutes and 2 hours. |
| 22. | Data saturation | Was data saturation discussed? | Data saturation was discussed after the third focus group. |
| 23. | Transcripts returned | Were transcripts returned to participants for comment and/or correction? | A summary of the findings of the focus groups was returned to participants for comment and/or correction. |
| **Domain 3: analysis and findings** | | | |
| Data analysis | | | |
| 24. | Number of data coders | How many data coders coded the data? | OJ and MG coded the data and EK was consulted for discrepancies. |
| 25. | Description of the coding tree | Did authors provide a description of the coding tree? | The mentioned topics were used in the coding tree. |
| 26. | Derivation of themes | Were themes identified in advance or derived from the data? | Themes were determined in advance of the data, but also derived from the data. The PAPM was applied during data analysis. |
| 27. | Software | What software, if applicable, was used to manage the data? | The data were analysed using NVivo version 12 software. |
| 28. | Participant checking | Did participants provide feedback on the findings? | Participants did not provide feedback on the findings. One participant showed his interest in the DobbelFit game after the focus group had taken place. |
| Reporting | | | |
| 29. | Quotations presented | Were participant quotations presented to illustrate the themes / findings? Was each quotation identified? e.g. participant number | Participant quotations were presented to illustrate the findings. All quotations were identified by participation number. |
| 30. | Data and findings consistent | Was there consistency between the data presented and the findings? | The research theme believes there was consistency between the presented data and the findings. |
| 31. | Clarity of major themes | Were major themes clearly presented in the findings? | The PAPM was used during data analyses and to identify major themes. The related topics were presented. |
| 32. | Clarity of minor themes | Is there a description of diverse cases or discussion of minor themes? | Diverse cases were described and also minor themes (e.g. opinions of single participants) were mentioned. |

*Abbreviations: Precaution Adoption Process Model (PAPM), Ellen Koster (EK), Marle Gemmeke (MG), Obaid Janatgol (OJ).*
